# Key Factors for the Emergence of Collective Decision in Invertebrates

**DOI:** 10.3389/fnins.2012.00121

**Published:** 2012-08-20

**Authors:** Raphaël Jeanson, Audrey Dussutour, Vincent Fourcassié

**Affiliations:** ^1^Centre de Recherches sur la Cognition Animale, Université de Toulouse, Université Paul SabatierToulouse, France; ^2^CNRS, Centre de Recherches sur la Cognition AnimaleToulouse, France

**Keywords:** collective decision, emergence, insect, invertebrate, non-linearity, self-organization, social interactions

## Abstract

In many species of group living invertebrates, in particular arthropods, collective decisions can emerge from the combined actions of individuals and the direct or indirect interactions between individuals. These decisions allow groups of individuals to respond quickly and accurately to changes that occur in their environment. Examples of such decisions are found in a variety of invertebrate taxa and in many different contexts, e.g., exploring a new territory, foraging for food, finding a suitable location where to aggregate or to establish a nest, defending oneself against predators, etc. In this paper we review the collective decisions that have been documented in different invertebrate taxa where individuals are known to live temporarily or permanently in social or gregarious groups. We first present some simple examples of collective decisions involving the choice between two alternatives. We then define the fundamental rules required for these collective decisions to emerge throughout the invertebrate taxon, from simple organisms such as caterpillars, to animals endowed with highly developed perceptive and cognitive capacities such as ants and bees. The presentation of these rules gives us the opportunity to illustrate one of the pitfalls of the study of collective choice in animals by showing through computer simulations how a choice between two alternatives can be misinterpreted as the result of the action of self-organized mechanisms. In the second part, we discuss the peculiarities of collective decisions in invertebrates, their properties, and characteristics. We conclude by discussing the issue of individual complexity in collective decision-making process.

## Historical Background

Ever since the first description of their behavior, from the classic naturalist literature to that of the mid twentieth century, invertebrates have been considered as simple organisms, endowed with limited cognitive capabilities. For example, despite detailed observations and clever experimental tests proving the contrary, the French entomologist Fabre (2000) clung to the belief that insects were unable to learn and were moved solely by instinct. Early behaviorists placed most invertebrates (with cephalopods as a notable exception) at the lower rungs of the ladder of animal intelligence and for a long time invertebrates were considered as the ideal model organisms for the study of behavioral reflex systems (Loeb, 1901; Kühn, 1919; Kandel, 2001). Yet, in the past 70 years, there has been a wealth of studies demonstrating that invertebrates, and particularly insects, are endowed with cognitive capabilities of the same level, or even superior, to those of many vertebrates. The impetus for these studies was certainly given by Karl von Frisch with his work on honeybees. The discovery that honeybees are able to learn flower locations, odors, colors, shape, and to communicate in an abstract way with their nestmates (Frisch, 1967) was the start of a huge research effort that allowed to unravel and to fully appreciate the complexity of the cognitive mechanisms underlying decision-making processes in bees (reviews by Menzel and Giurfa, 2001, 2006; Giurfa, 2007) and other insects (review by Menzel et al., 2007; Dornhaus and Franks, 2008; Wehner, 2009).

In many invertebrate species however, individuals do not live in isolation but form groups whose degree of sociality can be extremely variable, going from simple seasonal gathering of individuals at a favorable location (Waldbauer, 2001) to the highest form of sociality (Wilson, 1971; Hölldobler and Wilson, 1990, 2008; Costa, 2006; Seeley, 2010). Social insects in particular are made famous by their collective behaviors, as opposed to their individual performances. These collective performances are expressed in a variety of contexts, e.g., nest construction and maintenance, colony emigration, foraging, colony defense, and division of labor. For many years, scientists have sought to identify the “ghost in the machine” that gives rise to these collective performances but the explanations they provided were more of a poetic than of a scientific nature. The research that came later showed that collective performances of social insects were neither explained by a single all-powerful individual (termed “queen” by Réaumur) giving orders to her vassals, nor to a “spirit” (Maeterlinck, 1902) or a “soul” (Marais, 2009). At the beginning of the twentieth century, the great American myrmecologist Wheeler (1911) coined the term super organism to describe the structure and function of social insect societies. However, although appealing to biologists, this notion did little to further the understanding of the mechanisms allowing the transition from individual to collective performances in these insects. Wheeler (1928) later introduced the important notion of emergence in his book *The social insects: their origin and evolution*. He noted that “social insect colonies as a whole are not equivalent to the sum of their individuals but represent a different, emergent level,” Fifty years later, in the early 1980s, Jean-Louis Deneubourg, a student of Ilya Prigogine at the Free University of Brussels, was one of the first to give a reappraisal of Wheeler’s notion of emergence in social insects by including it within the framework of self-organization theory. Together with biologist Jacques Pasteels, they showed through computer simulations and mathematical models how social insect societies could be considered as complex systems in which the transition from the lower level components (individual workers) to the higher level component (colony) could be explained by self-organized mechanisms based on the use of simple rules by individuals relying solely on local information, and on the direct or indirect interactions among these individuals (Deneubourg et al., 1986; Pasteels and Deneubourg, 1987). So far this approach has proved to be extremely successful in accounting for a variety of behaviors observed at the collective level in social insects (review by Detrain and Deneubourg, 2006, 2008), as well as in other invertebrates (Table 1).

**Table 1:** **Collective decisions arising by self-organization mechanisms in binary choice experiments in invertebrates**.

Collective behavior studied	Taxon	Species	Type of information	Identical options	Different options	Reference
Choice of a shelter or of an aggregation site	Cockroaches	*Periplaneta* sp.	Tactile/chemical	Y	Y	Halloy et al. (2007), Sempo et al. (2009), Canonge et al. (2011), Leoncini and Rivault (2005), Said et al. (2005)
		*Blattella germanica*	Tactile/chemical	Y	Y	Ame et al. (2006), Rivault and Cloarec (1998), Ame et al. (2004, 2006), Jeanson and Deneubourg (2007)
	Isopods	*Porcellio scaber*	Tactile/chemical?	Y	Y	Devigne et al. (2011), Broly et al. (2012)
	Ants	*Messor barbarus*	Tactile/chemical	Y	Y	Jeanson et al. (2004a)
Choice of a new nest site	Ants	*Temnothorax* sp.	Tactile	N	Y	E.g., Pratt et al. (2002), Franks et al. (2002, 2003b, 2006)
		*Monomorium pharaonis*	Chemical	N	Y	Evison et al. (2012)
	Honeybees	*Apis mellifera*	Tactile/visual	Y	Y	Visscher (2007), Seeley et al. (2012), Seeley (2010)
Choice of a path during migration	Spiders	*Larinioides cornutus*	Silk (tactile/chemical)	Y	NA	Jeanson et al. (2004b)
		*Anelosimus eximius*	Silk (tactile/chemical)	Y	NA	Mailleux et al. (2008), Saffre et al. (1999)
Choice of a path during foraging	Annelids	*Eisenia fetida*	Tactile	Y	NA	Zirbes et al. (2010)
	Mites	*Dermatophagoides pteronyssinus*, *Tetranychus urticae*	Silk (tactile/chemical)	Y	Y	Yano (2008), Mailleux et al. (2010)
	Cockroaches	*Blattella germanica*	Chemical	Y	Y	Jeanson and Deneubourg (2006)
	Ants	*Linepithema humile*	Chemical	Y	Y	Goss et al. (1989), Vittori et al. (2006), Garnier et al. (2009)
		*Lasius niger*	Chemical	Y	Y	Beckers et al. (1992), Detrain et al. (2001), Dussutour et al. (2004, 2005, 2006)
		*Oecophylla longinoda*	Tactile	Y	NA	Lioni and Deneubourg (2004), Lioni et al. (2001)
Choice of a food source during foraging	Lepidopterans	*Malacosoma disstria*	Silk (tactile/chemical)	Y	Y	Dussutour et al. (2007), Dussutour et al. (2008)
	Cockroaches	*Malacosoma americanum*	Silk (tactile/chemical)	Y	Y	Fitzgerald (1995)
	Ants	*Blattella germanica*	Chemical	Y	NA	Lihoreau et al. (2010)
		*Lasius niger*	Chemical	Y	Y	Pasteels et al. (1987), Beckers et al. (1990, 1993), Portha et al. (2002)
		*Myrmica sabuleti*	Chemical	NA	Y	de Biseau et al. (1991)
		*Monomorium pharaonis*	Chemical	Y	Y	Sumpter and Beekman (2003)
		*Pheidole megacephala*	Chemical	Y	Y	Dussutour et al. (2009a)
		*Tetramorium caespitum*	Chemical/tactile	Y	Y	Collignon and Detrain (2010)
	Bees	*Apis mellifera*	Tactile	NA	Y	Seeley et al. (1991), Seeley (1995)
		*Trigona recursa*	Chemical	NA	Y	Schmidt et al. (2006)
		*Melipona fasciata*	?	NA	Y	Biesmeijer and Ermers (1999)
Choice of a path during exploration	Ants	*Linepithema humile*	Chemical	Y	NA	Deneubourg et al. (1990)
		*Lasius niger*	Chemical	Y	NA	Devigne and Detrain (2002)
		*Pheidole megacephala*	Chemical	Y	NA	Dussutour et al. (2009b)
Choice of two targets during colony defense	Bees	*Apis mellifera*	Visual/chemical	Y	NA	Millor et al. (1999)
Choice of two exits in a panic situation	Ants	*Atta insularis*	?	Y	NA	Altshuler et al. (2005)

*The type of choice and the behavioral context in which it occurs is indicated in the first column. The second and third column gives the taxon and the species in which the collective decision has been observed, respectively. The fourth column gives the nature of the information used by animals. ? means that no information has yet been provided on the type of information used. The fifth and sixth columns indicates whether experiments with choices between identical or different options have been performed (Y = choice observed, N = no choice observed, NA = experiments not performed or not reported)*.

## Definition and Examples of Collective Decisions in Invertebrates

### Definition of collective decisions

Self-organization allows a group of animals to make consensus decisions, i.e., to make a choice between two or more mutually exclusive alternatives without losing the cohesion of the group (Conradt and Roper, 2005; Sumpter and Pratt, 2009). Consensus decisions contrast with combined decisions in which individuals are influenced by each other but do not aim at reaching a unique decision. For the sake of convenience, we will solely be employing the term collective decisions from here on in. In some instances, the choice the group must make is critical for its survival, as when a swarm of honeybees chooses a cavity in which to settle (Seeley, 2010) or when a colony of house-hunting ants chooses a location in which to install its new nest (Franks et al., 2002). In most cases, although each member of the group only has access to partial information, and thus is unable to compare among the different alternatives offered, the properties of the mechanisms underlying these collective decisions are such that the best choice is made by the group, i.e., the choice of the highest quality food source (Beckers et al., 1990), the shortest path (Goss et al., 1989), or the best location at which to aggregate or settle a colony (Canonge et al., 2011). This has led to the notion of “swarm intelligence” (Bonabeau et al., 1999; Garnier et al., 2007; Blum and Merkle, 2010) and has been a source of inspiration for scientists working in other disciplines than biology, opening in particular new avenues of research in computer science (Ant Colony Optimization Algorithms: review by Blum, 2005) and robotics (swarm robotics: Pfeifer et al., 2007; Trianni, 2010).

A common misconception about collective decision-making is that it necessarily implies some sort of consultation among individuals within the group, the weighing of each other’s opinion, and the sharing of all the information available about all possible choices until a consensus is reached and all the members of the group adhere to a single decision. Although this type of collective decision can be found in non-human vertebrates (Conradt and Roper, 2005; Conradt and List, 2009), it is relatively rare in animals, particularly in invertebrates and, when present, always involves the intervention of self-organized mechanisms in the form of positive or negative feedbacks (Sumpter and Pratt, 2009). The reason for this lies in the fact that the species of invertebrates in which collective decisions arise generally form large groups and/or are distributed over a large and discontinuous space, e.g., nest chambers, which impedes communication between group members. It is worth noting that all individuals in the group have the same weight in the final decision, independent of the presence of one or several informed leaders in the group. By employing the word leaders we do not mean that the members of the group make allegiances to particular individuals; when informed leaders are present, their leadership character only lies in the fact that they possess more information than other members of the group at the start of the process and that they initiate the decision-making process. For example, consider the case of food recruitment in ants. When a scout ant has found a food source that it judges worth exploiting, it returns to the nest and simultaneously lays a pheromone trail. Once the scout has arrived in the nest, its nestmates are alerted either by the odor of the trail and/or by specific motor displays of the scout. In mass recruitment the action of the scout stops at this stage and recruited workers follow the trail until reaching the food source, while in group recruitment (de Biseau et al., 1994; Cerdá et al., 2009; Collignon and Detrain, 2010) recruited workers need to be guided by the scout. In both cases however, when several food sources are advertised at the same time, scouts have the same weight than other workers in the final decision as to which source is exploited. This also holds true for nest emigration in bees (Seeley and Visscher, 2004; Visscher, 2007; Sumpter and Pratt, 2009) and house-hunting ants (Franks et al., 2002; Sumpter and Pratt, 2009), at least as long as some scouts do not have prior knowledge of potential nest locations before the initiation of the emigration process. In the latter case, knowledgeable scouts can be disproportionally influential in the final decision (Stroeymeyt et al., 2011a).

### Examples of collective decisions in invertebrates

When a group of animals is offered a choice between two identical options, it randomly selects one alternative, which can lead over many replicates to a U-shaped distribution of choices. The emergence of such asymmetrical distributions in a uniform environment is a characteristic of collective decisions (Pasteels et al., 1987; Deneubourg and Goss, 1989; Camazine et al., 2001) and has been reported across various behavioral contexts and taxa (Table 1). In the following we give some examples of asymmetrical distributions observed in binary choice experiments in invertebrates.

**Figure 1:**
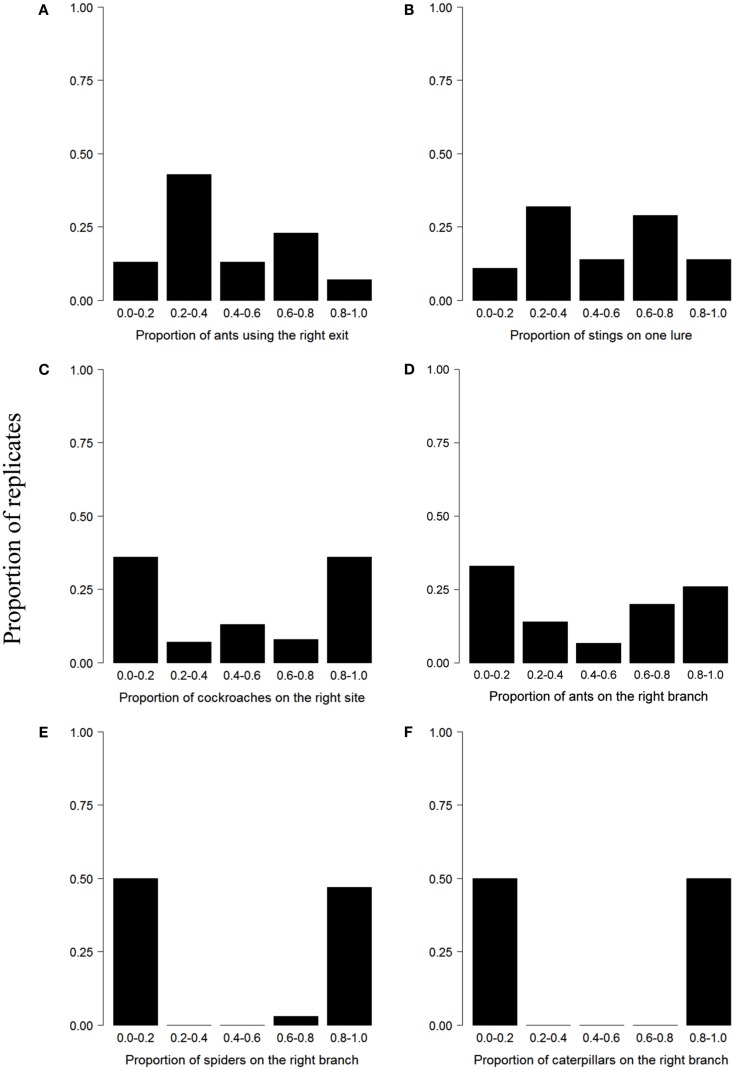
**Examples of U-shaped choice distributions across different behavioral contexts and taxa**. In all experiments, groups were faced with two identical options. **(A)** Selection of an exit during panic in the ant *Atta insularis* (30 replicates), adapted from Altshuler et al. (2005), **(B)** selection of a target by attacking honeybees *Apis mellifera* (31 replicates), from Millor et al. (1999), **(C)** selection of a shelter in the cockroach *Blattella germanica* (49 replicates), from Ame et al. (2004), **(D)** selection of a branch of a diamond-shape bridge in the ant *Lasius niger* (15 replicates), from Dussutour et al. (2005), **(E)** selection of an aggregation site in the spider *Larinioides cornutus* (30 replicates), from Jeanson et al. (2004b), **(F)** selection of a food source in the caterpillar *Malacosoma disstria* (20 replicates), from Dussutour et al. (2008). Published data or data provided by the authors were used to plot the histograms.

Figure 1A illustrates the collective behavior of ants in a panic situation. After being introduced in a circular arena with two similar exits, ants preferentially use a single door when a panic is induced by the addition of a strong repellent (Figure 1A). Figure 1B shows the collective defensive behavior of honeybees. When faced with two similar lures at the entrance of their hive, most bees focus their attack on one single target after their colony has been heavily disturbed (Figure 1B). The explanation lies in the fact that the barbed stingers left in place by honeybees emit an alarm pheromone that is attractive to nearby individuals. Initial fluctuations in the number of stings induce a small difference in the attractiveness of the targets that is amplified as the number of stings increased. Although bees preferentially focus their attacks on a single target, a second target can also be attacked (Figure 1B). This phenomenon is in part explained by the fact that some experiments are characterized by a low level of attacks, which prevents the initiation of an amplification process (Millor et al., 1999). Figure 1C represents the collective selection of a refuge by nymphs of the cockroach *Blattella germanica*. In presence of two identical shelters, groups of nymphs aggregate mostly at one site (Ame et al., 2004). In this case, amplification is mediated by a modulation of the individual resting time: the probability of leaving a shelter decreases with the number of conspecifics already present at the shelter. In the ant *Lasius niger*, foragers mostly use a single branch of a diamond-shaped bridge giving access to a food source from their nest (Figure 1D). This collective choice emerges from the trail-laying and trail-following behavior of the foraging workers. Small initial fluctuations of the relative concentration of pheromone on each branch are amplified by the successive passages of ants that eventually lead to the selection of a unique path. During migration, spiders lay down silk draglines that are attached discretly to the substrate. When they are given access to a bifurcated escape route, this pattern of silk attachment creates silk shortcuts that are followed by conspecifics (Figure 1E). In this system, the multiplication of silk strands laid by previous individuals serves as an amplification mechanism. When groups of tent caterpillars of the genus *Malacosoma* are offered a binary choice between two similar food sources, they massively exploit one resource and disregard the other (Figure 1F). During their displacement, caterpillars lay down silk threads impregnated with pheromones. The first caterpillar leaving a bivouac chooses a direction at random and the conspecifics that follow then reinforce the trail laid by the first individual (Dussutour et al., 2007). The similarity of the choice distributions observed for spiders and social caterpillars suggests that silk has very strong amplifying properties and that the initial activity of a few individuals is sufficient to give rise to clear-cut decisions. In these examples, the asymmetry of choices varies and depends notably on the strength of the underlying amplification mechanisms. In the following section, we will emphasize the contribution of feedbacks, the type of interactions, and noise in the emergence of collective decisions.

## Requirements of Collective Decisions

### Positive and negative feedbacks

**Figure 2:**
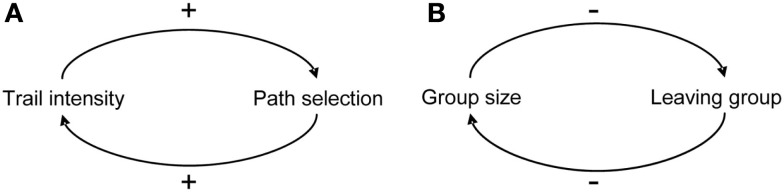
**Positive feedback loops in (A) the selection of one of two paths in ants (B) the selection of an aggregation site in cockroaches, “+” and “−” signs represent positive and negative influence respectively, from Jeanson and Deneubourg (2009) **.

The selection of a single option out of many alternative ones relies on the implementation of positive feedbacks or quorum response in which the probability of an individual of exhibiting a behavior is a non-linear function of the number of individuals already engaged in this behavior (Sumpter and Pratt, 2009). These feedbacks arise through a multitude of direct or indirect interactions among individuals and lead to amplification of random fluctuations (DeAngelis et al., 1986; Thomas, 1998; Jeanson and Deneubourg, 2009). Their contribution is critical for the expression of a clear-cut choice and the maintenance of the social cohesion of the group. They can be launched by the combination of positive interactions so that the change in the direction of the initial deviation is reinforced (Figure 2). For instance, the presence of a pheromonal trail increases the probability that an individual follows a path and reinforces it. Positive feedbacks can also arise from the combination of an even number of negative interactions. Hence the probability of leaving a group can decrease with group size and, consequently, favor large group formation. The action of positive feedbacks is generally counterbalanced by the existence of negative feedbacks that participate to the stabilization of emerging collective patterns (Camazine et al., 2001). For instance, crowding under a shelter in cockroaches or at a food source in ants, the exhaustion of a food source, and the existence of a limited number of foragers in a colony all constitute negative feedbacks. The emission of specific signals can also counteract positive feedbacks (Robinson et al., 2005). In honeybees for example, foragers experiencing attacks at a food source produce stop signals which causes the cessation of the waggle dances and thus decreases the recruitment to the food source under attack (Nieh, 2010). The same kind of signals are used during swarming by nest site scouts in order to inhibit other scouts from dancing and advertising other sites than their own. A mathematical model shows that this cross-inhibition between population of scouts advertising for different sites actually allows colonies to avoid potential deadlocks when they have to choose between two sites of equal quality (Seeley et al., 2012).

### Type of interactions involved in collective decisions and their impact

In order for positive feedbacks to function in social groups, it requires individuals to modulate their behaviors in response to interactions with conspecifics. In other words, the probability of an individual adopting a specific behavior depends on its ability to assess the number of conspecifics already engaged in that behavior or to detect traces of their earlier activities. This implies a direct or indirect exchange of information between group members.

Indirect interactions involve the perception of some trace of the earlier activities of conspecifics. Pheromonal trails in ants and silk strands in caterpillars are good examples of indirect interactions whose efficiency for the emergence of collective decisions depends upon their longevity in the environment. The latter can be strongly affected by abiotic factors and the physical structure of the environment. For instance, the persistence of trail pheromones strongly depends on ambient temperature or on the nature of the substrate on which they are deposited (Jeanson et al., 2003). In the ant *L. niger*, foragers usually select one branch of a diamond-shaped bridge connecting their nest to a foraging area (Figure 1D). However, in presence of two branches varying in their physico-chemical properties (quality of the paper covering each branch) ants preferentially follow the branch covered by the lighter paper (Detrain et al., 2001). The difference in the physico-chemical characteristics of the two substrates influences the accessibility of the pheromone trail to foragers and, through amplifying processes, leads to the choice of one branch over another. Therefore the characteristics of the environment can alter the outcome of collective decisions without altering individual behaviors.

On the other hand, direct interactions require the simultaneous presence of individuals. The best example of direct interactions in collective decision-making is given by the process called tandem-running that occurs at one stage of nest emigration in the ant *Temnothorax albipennis*. During tandem-running a single informed worker that knows of the location of a suitable new nest guides its nestmates to this location. The act of guiding is made through a tactile contact between the leader and the follower that keeps antennating the abdomen of the ant in front of him (Richardson et al., 2007). Tactile information also allows emigrating ants to assess the number of ants in a new nest and thus to judge if a quorum has been reached which determines the decision to stay in a candidate nest (Pratt, 2005). Direct interactions can also be mediated by odor as in cockroaches in which group formation relies on the perception of cuticular hydrocarbons (Rivault and Cloarec, 1998; Said et al., 2005). The combination of direct and indirect interactions, which are not mutually exclusive, can further enhance amplification giving rise to collective decisions.

### Noise

In non-linear systems, fluctuations at the individual level, even small ones, can lead to profound changes at the collective level, highlighting the fact that noise and stochasticity are intrinsic to any collective decision (Detrain and Deneubourg, 2008). For instance, in mass-recruiting ants, a well-known source of fluctuation or “noise” is related to the ability of foragers to faithfully follow a chemical trail. Recruited workers may lose the trail they follow or make “wrong” choices at trail junctions. When several food sources of identical quality are concurrently available in the nest surroundings, any slightly unbalanced distribution of workers and/or amount of trail marks over the different foraging paths leading to the food sources can lead the whole colony to select only one resource. The choice of one foraging path or one food source is therefore probabilistic and unpredictable. This has lead Deneubourg et al. (1983) to argue that noise or errors could be adaptive in the sense that they could offer ants the opportunity to discover a better alternative, e.g., a higher-quality food source or a shorter path. Therefore an optimal error level could exist which could minimize the time needed to discover better food sources and maximize foraging efficiency. It should be noted that noise may be induced by a large variety of sources in collective decision-making processes. Generally, the behavior of individuals never conforms exactly to the statistical average, rather, it exhibits variations over time, both within and between individuals. For example, both the amount of pheromone deposited per trip and the recruitment threshold, i.e., the amount of pheromone required to elicit the recruitment of a worker, may differ between individuals (Mailleux et al., 2000, 2003, 2005). Unlike “lost foragers,” such fluctuations do not directly favor the discovery of alternative sources. Instead, they simply introduce a small amount of variability (noise) into the decision-making process. Such undirected noise can be sufficient for the system to behave adaptively by facilitating quick transitions to more advantageous solutions in changing environments (Dussutour et al., 2009a). Similarly, in house-hunting ants, noise in the acceptability threshold of searching ants or in nest quality assessment by scouts during the selection of a new nest site allows flexibility and efficient decision (Marshall et al., 2006; Robinson et al., 2009).

The elucidation of the dynamics of collective decisions requires the characterization of the link between both the individual and collective levels. Modeling is a relevant approach because it allows to test whether the mechanisms that are predicted to act at the lower scale level (individuals) are able to generate the phenomenon observed at the level just above (group or colony; Camazine et al., 2001; Sumpter and Pratt, 2003). Models also aid in making predictions for conditions that are difficult to reproduce experimentally, e.g., large colony size in social insects or decision-making processes extending over a long time period. In the following section, we employ the modeling approach to emphasize the need to achieve experiments in which the group faces two identical alternatives.

## Modeling Collective Decisions

In order to illustrate the utility and benefit of the modeling approach in the study of collective decisions, we will employ a simple model developed in the context of shelter selection in cockroaches. An experimental group of first-instar *B. germanica* larvae were offered the choice between two identical resting sites (Ame et al., 2004). The empirical results indicated that cockroaches aggregated mostly at a single site after 24 h (Figure 1C). The authors identified a single behavioral rule that is sufficient to account for the choice pattern they observed: the individual probability of leaving a shelter decreases as the population in the shelter increases. This established, they went on to propose a straightforward mathematical model demonstrating that a collective decision can arise through this simple modulation. Formally, the individual probability *Q*_1_ of leaving shelter 1 as a function of the number of individuals *X*_1_ under shelter 1 is defined by:

(1) $$Q_{1}=\frac{\sigma }{k_{1}+X_{1}^{\eta }}$$

with *σ* = 0.06, *k*_1_ = 6, and η = 2. For values of η > 0, this function indicates that the probability of leaving a site decreases with the number of conspecifics *X*_1_ already at the site. The parameter η controls the steepness of the response to conspecifics, i.e., the degree of non-linearity: the higher the value of η, the greater the influence of conspecifics on the individual decision to move. The constant *k*_1_ represents the intrinsic attractiveness of shelter 1: the higher the value of *k*_1_, the lower the probability of leaving shelter 1.

**Figure 3:**
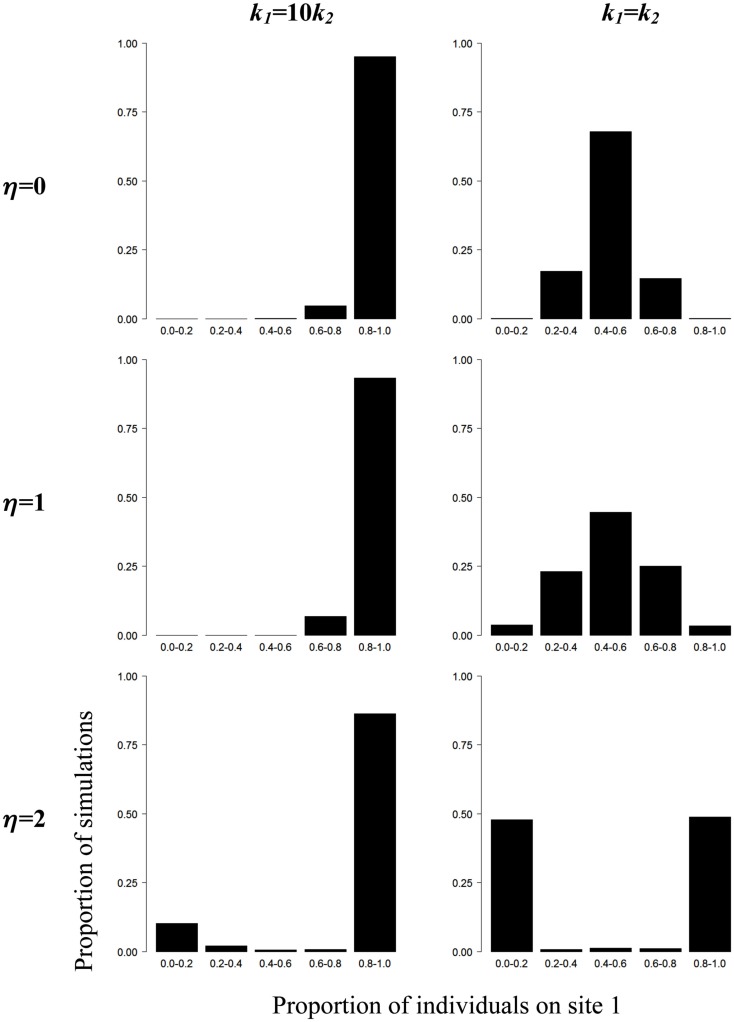
**Simulations of collective aggregation behavior at one of two sites in the cockroach**. Proportion of simulations (*n* = 1000) as a function of the proportion of individuals (*N* = 26) on shelter 1 in presence of two different (*k*_1_ = 10*k*_2_) or two identical (*k*_1_ = *k*_2_) sites and for different values of η (see text for details).

Using Monte Carlo simulations, we explore how variations in the values of η and *k* in Eq. 1 can influence the spatial distribution of individuals between shelters. In our simulations the values of η range between 0 and 2 and for η = 0, the individual decision to move does not depend on the presence of conspecifics, i.e., individuals behave as if they were alone. We also compare the collective patterns obtained by the simulations when groups of individuals are facing two identical (*k*_1_ = *k*_2_) or two different shelters (*k*_1_ = 10*k*_2_, i.e., the individual preference for shelter 1 is ten times greater than for shelter 2). At the beginning of each simulation run, individuals are randomly distributed between shelters. Then, at each time step, the individual probability of changing sites is determined by Eq. 1. An individual moves from one shelter to the other if a number drawn randomly between 0 and 1 is inferior or equal to *Q*_1_, otherwise it stays on its site. The individual probabilities *Q*_1_ and *Q*_2_ are updated at each time step as a function of the number of individuals *X*_1_ and *X*_2_ at each site. A thousand simulation runs with groups of 26 individuals are performed for each condition (time step: 1 s, simulation duration: 12 h). Each shelter can accommodate all individuals and cockroaches move immediately from one shelter to the other. The results are reported on Figure 3.

For *k*_1_ = 10*k*_2_ and values of η ranging between 0 and 2, the spatial distributions of individuals are qualitatively similar: the proportion of simulations as a function of the number of individuals at site 1 is highly right-skewed, i.e., most individuals aggregate at site 1. If one were looking at the spatial distribution of cockroaches without *a priori* knowledge about the underlying rules, one could erroneously conclude that a collective decision has arisen in all situations. However, our simulations show that the summation of the individual preferences for environmental heterogeneities (i.e., *k*_1_ = 10*k*_2_, η = 0) is sufficient to produce an asymmetrical distribution of cockroaches at the two sites, without the need to invoke the contribution of amplification processes.

Now, consider the situations where *k*_1_ = *k*_2_. For η = 0, individuals are evenly distributed between both sites, i.e., 50% of the population on average is found at each site. For η = 1, the individual decision to move depends on the presence of conspecifics but the strength of amplification is too weak to induce the collective selection of a single shelter. For η = 2 however, a dramatic change occurs: individuals strongly aggregate at a single site. The asymmetrical distribution observed provides strong evidence that a collective choice arose through interattraction and the implementation of positive feedback loops. A rigorous quantification of individual behaviors would then be required to identify the fundamental rules supporting amplification loops and driving the emergence of collective choice.

This model is a good illustration of the fact that binary choice experiments between two different alternatives are unable to provide insights into the mechanisms underlying a collective choice. In fact, without performing the crucial test where animals are given the choice between two strictly identical options it is impossible to know whether the asymmetric distribution of choice observed in tests with two different alternatives arises from social interactions between group members amplified by positive feedbacks or from the addition of individual responses to environmental heterogeneities. Only after having performed a test with two equal alternatives can one achieve experiments in which the group faces resources of different quality to disentangle the relative contribution of social interactions and individual responses in the collective decision observed.

## Peculiarities, Properties, and Characteristics of Collective Decision

### Influence of group size

In collective decision, the intensity of amplification processes, and thereby the degree of choice asymmetry, strongly depends on the number of individuals (or interactions) involved. For instance, small colonies of the ant *Monomorium pharaonis* cannot form efficient foraging trails because they do not generate high enough traffic to compensate for the evaporation of the trail pheromone (Beekman et al., 2001). Likewise, although ants offered a choice between two identical paths between their nest and a food source generally follow only one path (Beckers et al., 1992; Sumpter and Beekman, 2003; Dussutour et al., 2009b), models, and experiments show that when the flow of ants exiting the nest is too low the system is characterized by a unique unstable equilibrium in which both paths are used more or less equally. When the flow of ants exiting the nest increases a bifurcation occurs and the system reaches a stable equilibrium, with most ants using either the first or the second path. On the other hand, in the house-hunting ants *T. albipennis*, a collective decision can be reached even in small size colonies because of the peculiar mechanism used by ants when deciding to commit to a new nest. The workers will stay in a new nest only if a certain number of individuals, i.e., a quorum, have settled in that nest. This quorum however is not an absolute number but depends on the size of the colony so that a decision can be reached even in colonies containing less than 50 workers (the largest colonies of *T. albipennis* can contain more than 400 individuals; Dornhaus and Franks, 2006).

**Figure 4:**
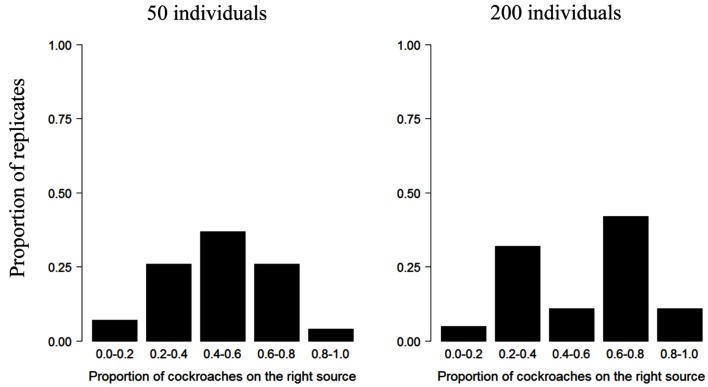
**Collective choice of one of two identical food sources in the cockroach *Blattella germanica***. Proportion of replicates of the experiment as a function of the number of cockroaches feeding at one of the two sources from Lihoreau et al. (2010), Lihoreau, Deneubourg, and Rivault (pers. com.). The asymmetry in the exploitation of the food sources is more pronounced in larger groups.

The influence of group size on collective decision is also illustrated by the experiments in which a group of cockroaches were made to choose between two food sources (Lihoreau et al., 2010) or two shelters (Ame et al., 2004). Whereas groups of 50 individual cockroaches exploit both food sources equally (50% individuals forage on each source), an asymmetry emerges in groups of 200 individuals, with the majority of individuals feeding on one of the food source only (Figure 4). From an experimental perspective, it is worth nothing that an absence of asymmetry in the exploitation of several resources does not necessarily imply that a group of animals is unable to achieve collective decisions; it could be simply explained by the fact that the conditions (e.g., critical group size) for them to emerge are not met.

It should be noted that the effect of group size on collective decisions strongly depends on the behavioral context in which they are expressed. In cockroaches, retention effects have been identified as responsible for both the selection of a shelter or the exploitation of a food source. Specifically, the average duration of feeding bouts increases with the number of individuals feeding at a food source (Lihoreau et al., 2010) and sheltering time increases with the number of individuals already present (Ame et al., 2004). In both situations, the probability of leaving a resource decreases with the number of individuals already present on it. However, the critical group size for the emergence of an asymmetrical choice is different in the two situations: although groups of 20 cockroaches are able to achieve a collective decision and select a single aggregation site, groups of 50 cockroaches are unable to choose between two equivalent food sources (Figures 1C and 4).

### Accuracy

In decision-making, speed and accuracy are often in opposition. Much time may be required to make an accurate decision between alternatives, because gathering, processing, and evaluating information may be a lengthy process. If an animal has to make a swift decision it may therefore be less discriminating. This link between speed and accuracy is so widespread that it has been termed the speed–accuracy trade-off paradigm (Busemeyer and Townsend, 1993; Osman et al., 2000; Franks et al., 2003a; Sumpter and Pratt, 2009; review by Chittka et al., 2009).

Although choosing collectively can lead to non-adaptive decisions in some notable exceptions (see Beckers et al., 1990 for the choice of a food source in foraging ants and Stroeymeyt et al., 2011b for the choice of a nest in house-hunting ants), on average it allows greater accuracy than do completely independent choice or weak responses to the behavior of conspecifics. The accuracy of groups of individuals in decision-making is typically predicted to be greater than that of isolated individuals; it initially increases with group size before leveling off (Krause et al., 2010). This phenomenon is driven by the fact that larger groups of individuals are more effective at gathering information than smaller groups or than solitary individuals, whereas the integration of the information gathered by different group members allows more accurate decisions to be made by larger groups (Couzin, 2009). Therefore collective decisions allow effective averaging of information without the need of complex comparison between options (Robinson et al., 2009).

The gathering of information prior to making a collective decision can be a way to circumvent the speed–accuracy trade-off. Hence in house-hunting ant *T. albipennis* colonies can gather and store information about available nest sites well before emigration, while their nest is still intact. This information is later retrieved and used during emigration and allows to improve simultaneously both speed and accuracy in the choice of candidate nests (Stroeymeyt et al., 2010).

Interestingly, steep threshold responses can sometimes amplify random fluctuations and lead to mass adoption of incorrect choices. This may lead animals in groups to make decisions that they would not make alone. In forest tent caterpillars for instance, isolated individuals show a high preference for a nutritionally balanced food source when offered the choice between a nutritionally balanced and a nutritionally unbalanced food source (Dussutour et al., 2007). In contrast, groups of caterpillars randomly choose one resource and are trapped at the first resource discovered. This results from an excessively strong and rapid amplification due to the silk laid down by caterpillars during foraging. While such amplification allows the maintenance of cohesion between group members, it prevents the group from achieving optimal diet choices.

### Robustness

Because distributed coordination does not depend on a specific subset of individuals, groups are inherently robust to perturbation (Camazine et al., 2001). Failure of one or several individuals usually does not put the group at risk. If an individual fails to carry out its task, another one promptly replaces it. Ants provide good examples of such robustness. Hence, in mass recruitment the removal of a scout ant does not affect recruitment since recruited workers “interact” mainly with the trail that has been laid by the scout and thus are kept continuously informed about the food location. Conversely, for decision based on direct interaction such as group recruitment, removing one individual from the population early in the decision process could have an important impact on the decision outcome because the mere presence of individuals is required to initiate the process. Worker interaction rates have been demonstrated to be robust to changes in group size or density (Pacala et al., 1996). Hitherto however, the robustness of collective decision in invertebrates has mainly been investigated through models (see e.g., Marshall et al., 2009).

### Flexibility

It is currently agreed upon that collective decisions lack flexibility, i.e., that many species in which collective decisions are observed are unable to adapt to dynamic environments, such as switching to exploiting a newly discovered high-quality food source when the foraging effort of the colony is already concentrated on a food source of lesser value (Beckers et al., 1990). The apparent inability of a group of animals to adapt to changing conditions is supported by laboratory experiments (Goss et al., 1989; Beckers et al., 1992; Traniello and Robson, 1995) and mathematical models (Goss et al., 1989; Nicolis and Deneubourg, 1999; Camazine et al., 2001). For example, in ants, pheromone trails allow a rapid collective choice for one alternative, but they also impose constraints on the overall foraging efficiency. Some ant species however are able to circumvent these constraints because of the properties of the trail pheromone they use. In particular, the decay rate of these pheromones plays an important role in the flexibility of collective foraging decisions: short-lived, volatile trails are more suited to the recruitment to ephemeral food sources because they can be rapidly modulated, whereas long-lived trails are more suited to the recruitment to persistent, or recurrent, food sources. When foraging in their natural environments, species of ant using a single pheromone trail experience a trade-off between efficient recruitment and flexibility in their response to the changes in the environment. For example, Goss et al. (1989) first provided Argentine ants (*Linepithema humile*) with a long path between their nest and a food source and after some time introduced a second, shorter path. When the short path was added after the ants had established their trail on the long path, the majority of ants continued to travel on the long path. Similar results have been reported with *L. niger* (Beckers et al., 1992). Mathematical models predict that ants will remain on an established trail for periods longer than the evaporation rate of the pheromone because ants continue to reinforce the trail on the long path (Goss et al., 1989; Nicolis and Deneubourg, 1999; Sumpter and Pratt, 2003). Pheromone trails can thus result in ants becoming “trapped” in suboptimal solutions.

That being said, there are ways to escape the deadlocks of suboptimal solutions however. For example, theoretical models on food recruitment usually consider that ants use just one single trail pheromone (e.g., Pasteels et al., 1987; Nicolis and Deneubourg, 1999); in practice however, many species of ants use a variety of pheromones to mark the path to food discoveries (Jeanson et al., 2003; Wyatt, 2003; Jackson et al., 2007) and the interplay of two pheromones has been demonstrated to be important under dynamically changing foraging conditions (Dussutour et al., 2009a; Reid et al., 2011). This is the case of the ant species *Pheidole megacephala* which uses two different pheromones, a long-lasting pheromone during exploration and a short-lasting exploitation pheromone during recruitment to a food source. Theoretical models and experiments indicate that the combination of these two pheromones allows *P. megacephala* colonies to track changing foraging conditions more effectively than would a single pheromone. When colonies of this species were provided first with a long path between their nest and a food source and then with a shorter path after some time, the majority of ants were able to select the short path, even if ants had already established a chemical trail on the long branch (Dussutour et al., 2009b). In the same way, species using positive feedback loops channeled by direct interactions such as tandem-running may be more flexible than species using mass pheromone recruitment and may prevent the colonies from locking to poor choice. For example, *T. albipennis* colonies are able to correct errors by continuing to survey potential nest sites during the last stage of the emigration process or even after they have settled altogether in a new nest (Dornhaus et al., 2004; Franks et al., 2007). If a nest site of better quality is discovered ants are able to switch nest.

### Collective decisions and individuality

Many studies on collective decision-making processes consider that social groups are composed of identical and interchangeable individuals. This assumption has proved to be valid in many contexts and has provided relevant insights for the understanding of the global dynamics and properties of the systems under study. Under some circumstances however, considering inter-individual variability can further improve our comprehension of whole-group functioning. For instance, individuals in groups of animals can differ in sex, body size, and/or the morphological/temporal caste they belong to. This may lead to differences in their response threshold to the signals involved in collective decisions, e.g., trail pheromone (Detrain and Pasteels, 1991; Morgan et al., 2006; Kleineidam et al., 2007). Groups can also contain individuals with differing behavioral tendencies, i.e., “personalities” (Sih et al., 2004, caterpillars: Dussutour et al., 2008; Nicolis et al., 2008; bumblebees: Burns, 2005, spiders: Pruitt and Riechert, 2011, honeybees: Burns and Dyer, 2008; ants: Chapman et al., 2011). Inter-individual differences may have important consequences for collective decisions; for instance, these differences have been shown to lead to colony decisions that are dependent upon the ratio of the different categories of individuals in the group. Dussutour et al. (2008) have shown that in social caterpillars, individuals within a group fall into two clearly distinguishable behavioral categories: inactive and active. Active caterpillars spend considerable time exploring the environment and relatively little time feeding, whereas inactive caterpillars have longer meals and explore less. At the collective level, when given a choice between two equal low quality food sources, active caterpillar-biased colonies are less cohesive than colonies comprised of proportionately fewer active caterpillars. They do not focus their activity on one source but split and exploit two sources at the same time. In contrast, inactive caterpillar-biased colonies focus their activity on one source only. In the case of social caterpillars collective behavior patterns can thus be explained by individual differences. By the same token, social insect colonies can benefit from having workers with different behavioral types. For example, studies on foraging honeybees show that co-existing strategies, where some individuals place more emphasis on accuracy and others on speed, can be advantageous to the colony in a variable environment (Burns and Dyer, 2008). The importance of inter-individual differences in collective decisions has not been fully investigated. Yet, individuality is recognized as an intrinsic character of all biological systems in which no two individuals are the same. This question thus appears critical for the understanding of collective behaviors and collective decisions in animals, including invertebrates.

### Collective decisions and individual complexity

Studies on collective decisions generally assume that complex structures or behaviors at the collective level can be explained by simple behavioral rules at the individual level. This issue has been the subject of some misinterpretation in the early history of the studies of self-organization in collective behaviors (Deneubourg et al., 1999; Camazine et al., 2001) and to the rejection of this approach by some biologists. It should be emphasized, however, that the self-organization approach does not deny individual complexity, particularly from a cognitive point of view. In fact, the degree of individual complexity found in animal groups can be extremely variable and even the tenant of self-organization will acknowledge that individual complexity can also be a source of collective complexity (Anderson and McShea, 2001). Self-organization studies simply apply a principle that is universally used in science: that of parsimony. There is no need to invoke the contribution of complex individual behaviors when models of collective decisions show that simple behavioral rules are able to account for the production of complex collective patterns. For instance, the selection of the more rewarding of two food sources in ants does not require any active comparison between the food sources at the individual level but instead only relies on a modulation of the trail-laying behavior of individual ants in response to the quality of the food sources. In the same way, the modulation of individual behavior (staying or leaving) by cockroach nymphs as a function of the number of conspecifics in the neighborhood when aggregating at a single site does not require that these insects explicitly count the number of individuals in their surroundings. To do so they may just rely on an assessment of the overall quantity of aggregation pheromones (i.e., cuticular hydrocarbons) perceived at a site, which is a function of both the number and the sex of the individuals in their surroundings (Jeanson and Deneubourg, 2007). Of course, this does not mean that insects are unable to make direct and subtle comparisons between alternatives, as has been reported for a long time in parasitoids (Wajnberg et al., 2007) or more recently in house-hunting ants (Sasaki and Pratt, 2011). Moreover, complex cognitive processes such as learning and memory can also be involved in collective decisions. Private navigational information (memory) can either override social information (trail pheromone in ants or waggle dance in honeybees) and thus reduce the flexibility of collective decisions by counteracting amplification process (Grüter et al., 2008, 2011) or, on the contrary, it can contribute to enhance the amplification process (trail-following behavior in ants: Czaczkes et al., 2011). For example, workers of the ant *L. niger* are able to memorize rapidly the spatial location of a food source on the basis of the visual cues they find in their surroundings (Aron et al., 1993; Evison et al., 2008; Czaczkes et al., 2011; Grüter et al., 2011). Initially, recruited workers follow the chemical trail laid down by their nestmates, but they are rapidly able to orient on the sole basis of the visual cues they have memorized. In red wood ants, such a process leads foraging workers to develop a fidelity to a particular trail (Rosengren, 1971) and, on a longer time scale, explains the stability of the network of foraging trails around their nest over successive years (Rosengren and Fortelius, 1986; Salo and Rosengren, 2001). On the other hand visual memory can also act as a constraint that restrains amplification processes and thus limits the flexibility allowed by this latter when rapid changes occur in the localization of the resources exploited (Fewell, 1990; Grüter et al., 2008; Grüter and Ratnieks, 2011). Memory may also play an important role in house-hunting ants. Knowledgeable workers that had the possibility to gather information about potential nest sites prior to the emigration event allow to enhance the collective performance of the colony during the choice of a new nest and play a disproportionate role in this decision (Stroeymeyt et al., 2011a).

A last detail that needs to be emphasized is that although the behavioral rules at the basis of collective decisions can be simple, it is rarely true of the underlying mechanisms (physiological, neural, sensorial, perceptual, or cognitive) that allow these rules to be expressed (Seeley, 2002). For example, the statement that dancing in bees or pheromone deposits in ants are simple behaviors does not imply that complex mechanisms occurring upstream the behavioral performance are not involved. In ants, a scout that has discovered a food source must integrate several parameters before deciding if and how it will recruit nestmates to the food source. These parameters depend on both the characteristics of the food source (quality, novelty, accessibility, transportability; review by Detrain and Deneubourg, 2002) and on the nutritional needs of its colony (Mailleux et al., 2006). In the same way, in the context of nest-moving in honeybees, a scout’s decision to perform a waggle dance for recruiting additional scouts to a cavity depends on its capacity to assess and weigh multiple parameters, e.g., the cavity volume, shape or temperature, and its exposure to sunlight or wind (Seeley, 1977, 2010; Seeley and Morse, 1978).

## Conclusion

The ability to organize collectively and to make collective decisions is generally assumed to be the hallmark of highly social species. Our review shows however that collective decisions in invertebrates are not only found in social insects, such as ants or bees, but also in other organisms that, at least at some point in their life cycle, show some form of sociality. Generally, collective decisions immediately benefit all individuals in a group by allowing the rapid selection of the best of several different options. Whether this ultimately can lead to increase their fitness depends on a variety of factors related to the organisms’ biology and their environment. In ants for example, in which the greatest part of the colony is constituted by sterile individuals that share a common interest, the ability to use a collective exploration strategy and to choose and monopolize the best among several food sources through chemical mass recruitment have probably been determinant in the evolution of the dominant character of some species. In fact, both of these features happen to be common to most invasive ant species (Lach et al., 2010). In other invertebrate organisms, collective decisions may not always be ultimately advantageous and are probably subject to a cost-benefit trade-off, much alike that which has been discussed in behavioral ecology for group living (Krause and Ruxton, 2002). For example, the collective choice of a single favorable site at which to aggregate in cockroach can be advantageous on the short-term but can lead on the long-term to an increase in the competition for food (which can generate cannibalism) or to the rapid spread of potentially lethal pathogens. In any case, collective decisions have so far been studied more from a mechanistic than from a functional point of view and, although collective decisions are generally assumed to benefit animals, their functional properties would certainly deserve to be investigated in dedicated studies in order to understand the selective forces acting on them (Boomsma and Franks, 2006). This can be achieved only by examining the outcome of collective decisions in the natural environment of the species under study (Traniello and Robson, 1995).

One of the objectives of this review was to highlight the importance of comparative studies for the study of collective decisions in invertebrates. Studies on different organisms allow to delineate the generic rules underlying collective decisions and the key factors required for their emergence. They could also be useful in developing scenarios for the evolutionary steps that have lead to the emergence of collective decisions within some groups of invertebrates like ants or bees in which they play a major role for the cohesion of the colonies. It appears that one of the key factors required for the emergence of collective decisions is the ability of an individual to modulate its behavior as a function of the quality of the resource it has found (e.g., trail-laying in ants) or as a function of its conspecifics’ behavior (e.g., staying at a site or leaving it in cockroaches). It could thus be predicted that any species of invertebrates in which individuals are endowed with such capabilities would potentially be able to make collective decisions. Further studies on a more extended range of invertebrate organisms will allow to investigate whether this prediction is true or not.

## Conflict of Interest Statement

The authors declare that the research was conducted in the absence of any commercial or financial relationships that could be construed as a potential conflict of interest.
